# High-Mobility Group Nucleosome-Binding Protein 1 as Endogenous Ligand Induces Innate Immune Tolerance in a TLR4-Sirtuin-1 Dependent Manner in Human Blood Peripheral Mononuclear Cells

**DOI:** 10.3389/fimmu.2018.00526

**Published:** 2018-03-14

**Authors:** Rob J. W. Arts, Po-Kai Huang, De Yang, Leo A. B. Joosten, Jos W. M. van der Meer, Joost J. Oppenheim, Mihai G. Netea, Shih-Chin Cheng

**Affiliations:** ^1^Department of Medicine, Radboud Center for Infectious Diseases, Radboud University Medical Center, Nijmegen, Netherlands; ^2^College of Life Science, Institute of Molecular Medicine, National Tsing Hua University, Hsinchu City, Taiwan; ^3^Cancer and Inflammation Program, Center for Cancer Research, National Cancer Institue at Frederick, Frederick, MD, United States; ^4^Human Genomics Laboratory, Craiova University of Medicine and Pharmacy, Craiova, Romania

**Keywords:** high-mobility group nucleosome-binding protein 1, endotoxin tolerance, sterile inflammation, sirtuin-1, macrophages

## Abstract

High-mobility group nucleosome-binding protein 1 (HMGN1) functions as a non-histone chromatin-binding protein in the cell nucleus. However, extracellular HMGN1 acts as an endogenous danger-associated inflammatory mediator (also called *alarmin*). We demonstrated that HMGN1 not only directly stimulated cytokine production but also had the capacity to induce immune tolerance by a TLR4-dependent pathway, similar to lipopolysaccharide (LPS)-induced tolerance. HMGN1-induced tolerance was accompanied by a metabolic shift associated with the inhibition of the induction of Warburg effect (aerobic glycolysis) and histone deacetylation *via* Sirtuin-1. In addition, HMGN1 pre-challenge of mice also downregulated TNF production similar to LPS-induced tolerance *in vivo*. In conclusion, HMGN1 is an endogenous TLR4 ligand that can induce both acute stimulation of cytokine production and long-term tolerance, and thus it might play a modulatory role in sterile inflammatory processes such as those induced by infection, trauma, or ischemia.

## Introduction

High-mobility group (HMG) proteins are non-histone nuclear proteins. They bind to nucleosomes and regulate chromosome architecture and gene transcription (1). However, upon cell stimulation or under stress situations, such as mechanical change and tissue damage, HMG proteins can be either released or excreted into the extracellular space (2). HMGB1 is the best-characterized HMG-family protein: it is released from injured or activated innate immune cells (1), it stimulates cytokine and chemokine production (3), it can induce dendritic cell activation (4), and it is chemotactic and functions an alarmin.

High-mobility group nucleosome-binding protein 1 (HMGN1) belongs to the HMG N family but it exhibits no homology to HMGB1. The functions of HMGN1 were mainly related to its nuclear localization, including modulating histone phosphorylation (5, 6), acetylation (7), methylation preferentially at CpG island-containing promoters (8, 9), and enhancement of DNA damage repair (10). However, two recent studies showed that HMGN1 also has a biological role as an alarmin by inducing DC maturation, antigen-specific immune responses, and antitumor immunity (11, 12).

Upon engaging microbial or endogenous ligands, innate immune cells either directly clear them by phagocytosis, or they induce production of cytokines and chemokines for further activation of the immune system. After the acute inflammatory phase, a resolution phase is actively induced in order to limit the potentially deleterious ongoing inflammation, followed by a return to steady state. Thus, after the initial marked inflammatory response [e.g., induced by the Gram-negative cell wall component lipopolysaccharide (LPS)], subsequent re-stimulation of leukocytes is no longer able to induce the release of inflammatory mediators, but instead activates anti-inflammatory and repair proteins, a process termed innate immune tolerance (13, 14). Interestingly, the first exposure of monocytes to other microbial stimulants “trains” or “primes” the cells and they respond in a more robust way to a secondary stimulation or infection (15).

We have hypothesized that the first exposure of the innate cells to HMG proteins may also induce their functional reprogramming resulting in either tolerance or training. We showed that HMGN1 functions as an endogenous TLR4 ligand that, on the one hand, stimulates acute cytokine production and, on the other hand, induces tolerance in monocytes through a Sirtuin-1-dependent mechanism.

## Materials and Methods

### Isolation and Stimulation of Peripheral Blood Mononuclear Cells (PBMCs)

Separation and stimulation of PBMCs was performed from buffy coats obtained from healthy blood donors after written informed consent (Sanquin Bloodbank, Nijmegen). PBMCs were adjusted to a concentration of 5 × 10^6^ cells/ml and incubated at 37°C in flat-bottom 96-well plates (100 μl/well) with either 100 ng/ml HMGB1, 100 ng/ml HMGN1, 10 ng/ml LPS (E. coli strain O55:B5, Sigma Chemical Co., St. Louis, MO, USA), or culture medium. Recombinant HMGN1 was produced using an insect expression system constructed as previously reported (5). HMGN1 in the culture supernatant of High Five insect cells was purified under sterile condition by affinity chromatography. The endotoxin level in our HMGN1 preparation is <0.02 EU per μg of protein as determined by Pierce LAL Chromogenic Endotoxin Quantitation Kit (Cat #88282). To assess direct stimulation of cytokines, supernatant was removed and stored for assessment.

To study the potential reprogramming effects of HMGN1 on the function of monocytes/macrophages, after the initial stimulation for 24 h the cells were washed with warm PBS, allowed to rest for 24 h in RPMI containing 10% pooled human serum, and then restimulated with LPS (10 ng/ml), Pam3Cys (10 µg/ml, EMC microcollections, Tuebingen, Germany), flagellin (2 µg/ml, Sigma), or co-culture of Pam3Cys and *Candida albicans* β-1,3-(d)-glucan [10 µg/ml, kindly provided by D. Williams (East Tennessee State University)] for an additional 24 h. For the long-term studies, cells were incubated for a period of 6 days after the initial 24 h exposure to HMG proteins or LPS. On day 7, the cells were restimulated with the same stimuli for additional 24 h. Supernatants were collected 24 h after restimulation and stored at −20°C.

The receptor pathways involved in the biological effects of HMGN1 were assessed by blocking TLR4 with the natural antagonist *Bartonella quintana* LPS (16). A potential role for histone methylation or acetylation in the long-term effects of HMGN1 was assessed using specific pharmacological inhibitors: ITF2357 (100 nM, Histone deacetylase inhibitor, ITALFARMACO S.p.A, Milano, Italy), EGCG (30 µM, Epigallocatechin-3-gallate, histone acetyltransferase inhibitor, Sigma), and pargyline (3 µM, histone demethylase inhibitor, Sigma) or EX527 (10 µM, sirtuin-1 inhibitor, Sigma) (15, 17).

### Animal Experiments

Female C57BL/6J mice (8–10 weeks old, weighing 20 ± 3 g) were obtained from National Laboratory Animal Center (Taipei, Taiwan). All mice were housed in a pathogen-free facility. Animal welfare and experimental procedures were carried out in accordance with the National Institute of Health Guide for the Care and Use of Laboratory Animals, with the approval of the Institutional Animal Care and Use Committee of National Tsing Hua University (Approval number: 10530, Hsinchu, Taiwan). Mice were treated with PBS, recombinant HMGN1 (10 µg per mice) or E. Coli LPS (20 µg per mouse) by intraperitoneal injection. A second injection of LPS (20 µg per mouse) was performed after 6 h post first injection intraperitoneally. Blood samples were collected 1 h post second LPS injection for serum cytokine determination.

### Cytokine and Lactate Measurements

IL-6, IL-8 (Sanquin, Amsterdam, Netherlands), TNF-α, IL-1β (R&D, the Netherlands) concentrations in the culture supernatant were measured by commercial ELISA kits. The lowest detection limits are 0.78, 0.78, 3.9, and 3.9 pg/ml for IL-6, IL-8, TNF-α, and IL-1β, respectively. Mouse serum cytokine were measured by Cytokine Beads Array (Becton Dickinson, NJ, USA) according to the manufacturer’s instructions. Lactate was measured by a Lactate Fluorometric Assay Kit (Biovision, CA, USA). Delta lactate production (lactate concentration in the LPS restimulated sample minus the RPMI restimulated sample) is depicted in the figures.

### mRNA Extraction and RT-PCR

Cells were primed with either HMGN1 or LPS and restimulated with LPS as described above. mRNA was extracted by Trizol 4 h post-stimulation. The qPCR primers sequence are listed in the (Table S1 Supplementary Material) For sirtuin-1 expression, cells were stimulated for 4 h before RNA was isolated. cDNA was synthesized from 1 µg of total RNA by use of SuperScript reverse transcriptase (Invitrogen). Relative mRNA levels were determined using the Bio-Rad i-Cycler and the SYBR Green method (Invitrogen). Values are expressed as fold increases in mRNA levels, relative to those in unstimulated cells, with HPRT as a housekeeping gene.

### Statistical Analysis

Results from at least three sets of experiments were pooled and analyzed using GraphPad Prism software. Data are given as means + SEM and the paired Wilcoxon test or one-way ANOVA was used to compare differences between groups. The level of significance was set at *p* < 0.05.

## Results

### HMGN1 Induces Pro-Inflammatory Cytokine Production in PBMCs

**Figure 1:**
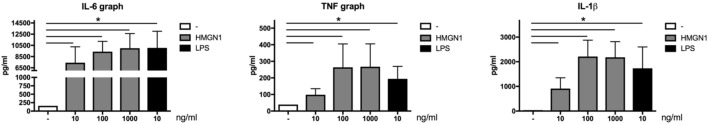
High-mobility group nucleosome-binding protein 1 (HMGN1) induces pro-inflammatory cytokine production. Human peripheral blood mononuclear cells were stimulated with recombinant HMGN1 or lipopolysaccharide (LPS) in a dose-dependent manner. HMGN1 concentration used were 10, 100, and 1,000 ng/ml, and LPS concentration was 10 ng/ml. Supernatant was harvested after 24 h stimulation. IL-6, TNF-α, and IL-1β production was determined by ELISA (*n* = 6).

We first examined the capability of HMGN1 to induce pro-inflammatory cytokine production in human PBMCs. HMGN1-induced considerable IL-6, TNF-α, and IL-1β production in PBMCs after 24 h stimulation in a dose-dependent manner (Figure 1). Strikingly, HMGN1 at 100 ng/ml could induce comparable amount of IL-6 and TNF-α and more IL-1β compared to that induced by LPS at 10 ng/ml.

### HMGN1 Induces Immune Tolerance in PBMCs

**Figure 2:**
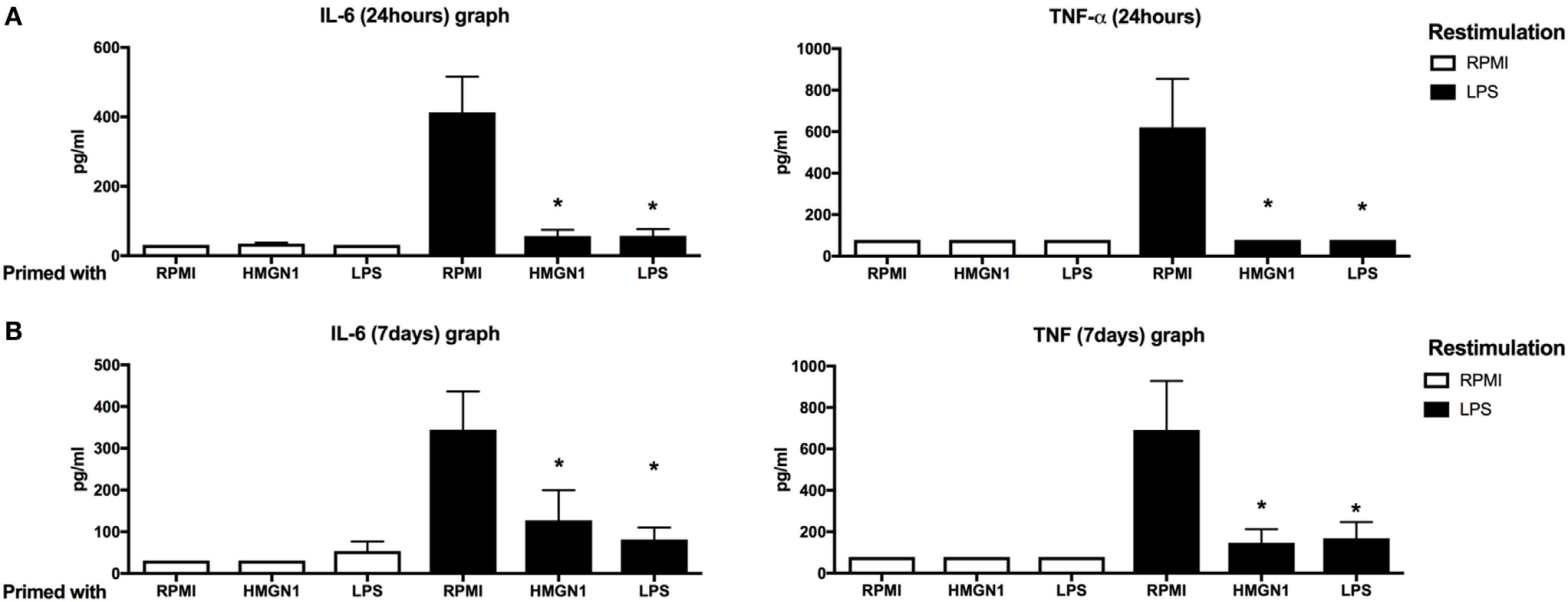
High-mobility group nucleosome-binding protein 1 (HMGN1) induces short- and long-term immunotolerance. Human peripheral blood mononuclear cells were primed with recombinant HMGN1 or lipopolysaccharide (LPS) for 24 h and then washed with PBS. The cells were further rested in RPMI containing 10% serum for **(A)** 24 h or **(B)** 6 days, and then restimulated with LPS or RPMI for additional 24 h and supernatant was harvested. The IL-6 and TNF-α level were determined by ELISA [*n* = 8, *p < 0.05 vs RPMI (LPS restimulated) control].

We hypothesized that HMGN1 may induce long-term effects on innate immune cells. To assess this possibility, PBMCs were first stimulated with HMGN1 or LPS (as a positive control). After 24 h stimulation, cells were washed with PBS to remove remaining stimulants and rested for an additional 24 h or 6 days, before secondary LPS stimulation was performed. IL-6 and TNF-α production upon secondary LPS (TLR4 ligand) stimulation were significantly impaired in HMGN1 pretreated monocytes both in short-term (Figure 2A) and long-term (Figure 2B) tolerance models, suggesting HMGN1-induced considerable tolerance against LPS stimulation. The HMGN1-induced tolerance is similar to LPS-induced tolerance.

### HMGN1 Induces Tolerance to Different TLR Agonists in PBMCs

**Figure 3:**
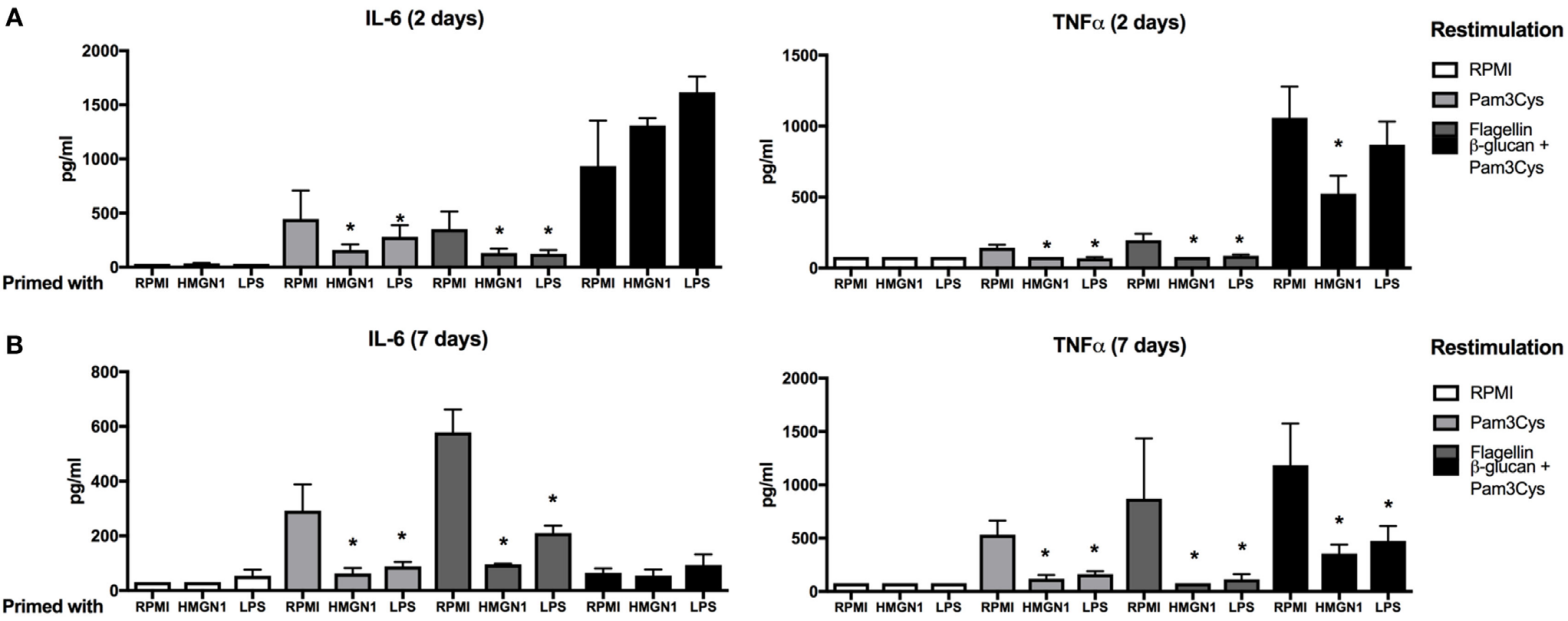
High-mobility group nucleosome-binding protein 1 (HMGN1) immunotolerance is not specific for TLR4 ligands. Human peripheral blood mononuclear cells were primed with recombinant HMGN1 or lipopolysaccharide (LPS) for 24 h and then washed with PBS. The cells were further rested in RPMI containing 10% serum for **(A)** 24 h or **(B)** 7 days then stimulated with Pam3Cys, flagellin, β-glucan, or RPMI, respectively, for additional 24 h and supernatant were harvested. The IL-6 and TNF-α levels were determined by ELISA (*n* = 4–8 *p < 0.05 vs RPMI control within each group of restimulation).

To further examine whether the HMGN1-induced tolerance is specific for TLR4 ligands or more general for other microbial ligands as well, we extended the study by also using TLR2 and TLR5 agonists, as well as the dectin-1 ligand β-glucan. We found that both HMGN1 and LPS could induce partial cross-tolerance to other TLRs both in short-term (Figure 3A) and long-term (Figure 3B) experiments. Only a partial tolerance effect was induced on dectin-1-induced TNFα production.

### Blocking TLR4 Signaling Attenuates HMGN1-Induced Tolerance

**Figure 4:**
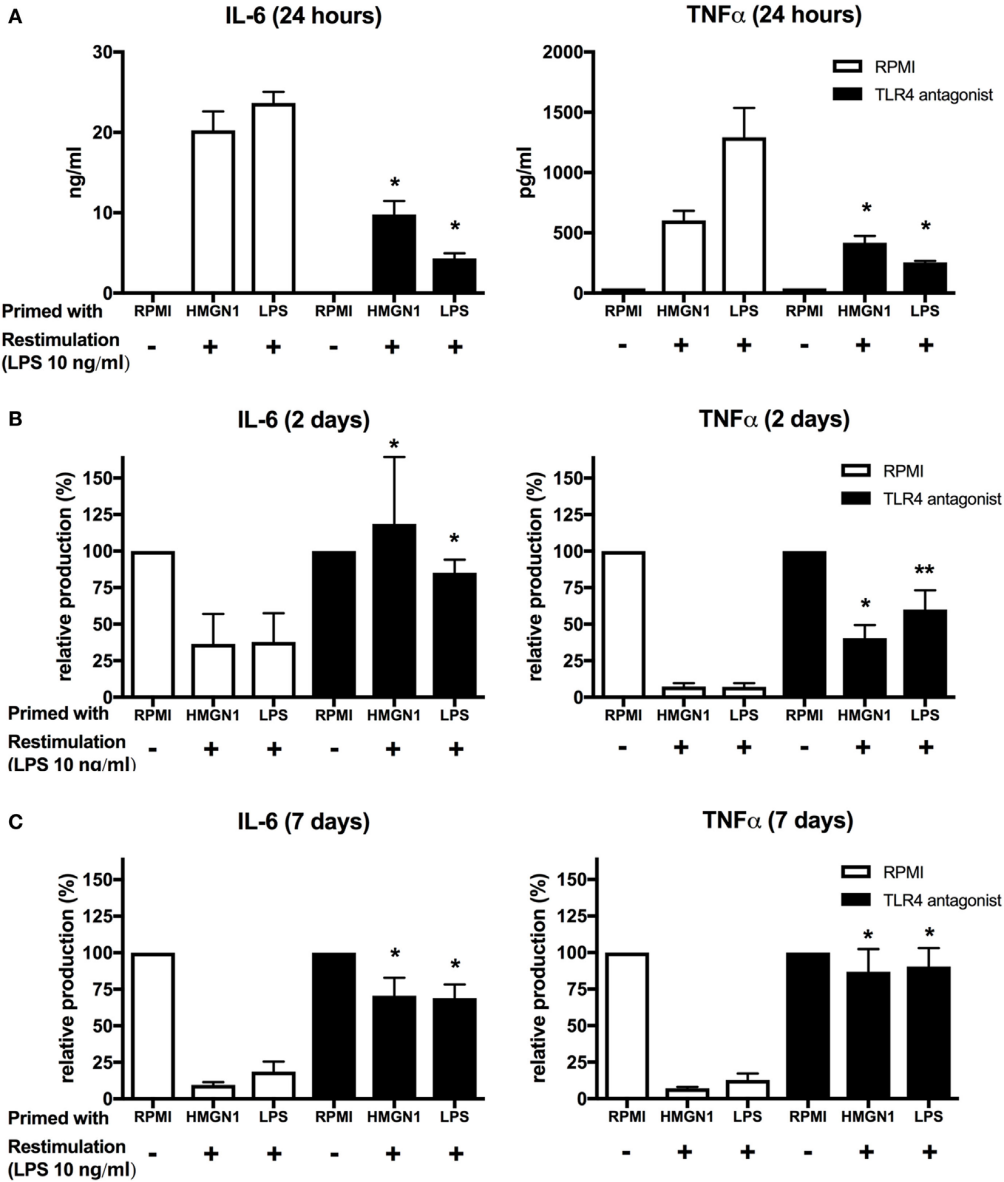
High-mobility group nucleosome-binding protein 1 (HMGN1) immunotolerance is dependent on TLR4 ligation. **(A)** Human peripheral blood mononuclear cells were preincubated with *B*. *quintanea* lipopolysaccharide (LPS; TLR4 antagonist) for 1 h and then primed with recombinant HMGN1 or LPS for 24 h. Supernatant was then harvested for IL-6 and TNF-α measurement. The cells were further rested in RPMI containing 10% serum for **(B)** 24 h or **(C)** 7 days and then stimulated with LPS or RPMI for additional 24 h. Supernatant was harvested after 24 h stimulation. The IL-6 and TNF-α level were determined by ELISA. The relative production of IL-6 and TNF-α compared to the RPMI control was presented (*n* = 4–8 *p < 0.05 vs RPMI control).

It has been suggested that HMGN1-induced dendritic cell maturation *via* TLR4 (11, 12). Therefore, we examined whether TLR4 is involved in HMGN1-induced tolerance in PBMCs. To block TLR4 signaling, PBMCs were first incubated with *B. quintana* LPS, a natural antagonist of TLR4 (16) for 1 h, followed by stimulation with HMGN1 or LPS. Pretreatment of cells with *B. quintana* LPS resulted in markedly reduced production of IL-6 and TNF-α upon LPS stimulation and a partial reduction upon HMGN1 stimulation (Figure 4A). Thereafter, we assessed both the short- and long-term tolerance effects induced by HMGN1. TLR4 blockade by antagonists blocked or reversed the tolerance effects induced by HMGN1 on IL-6 and TNF-α production (Figures 4B,C).

### HMGN1-Induced Tolerance Is Restricted to Pro-Inflammatory Cytokines, But Not to the Antimicrobial Peptides

**Figure 5:**
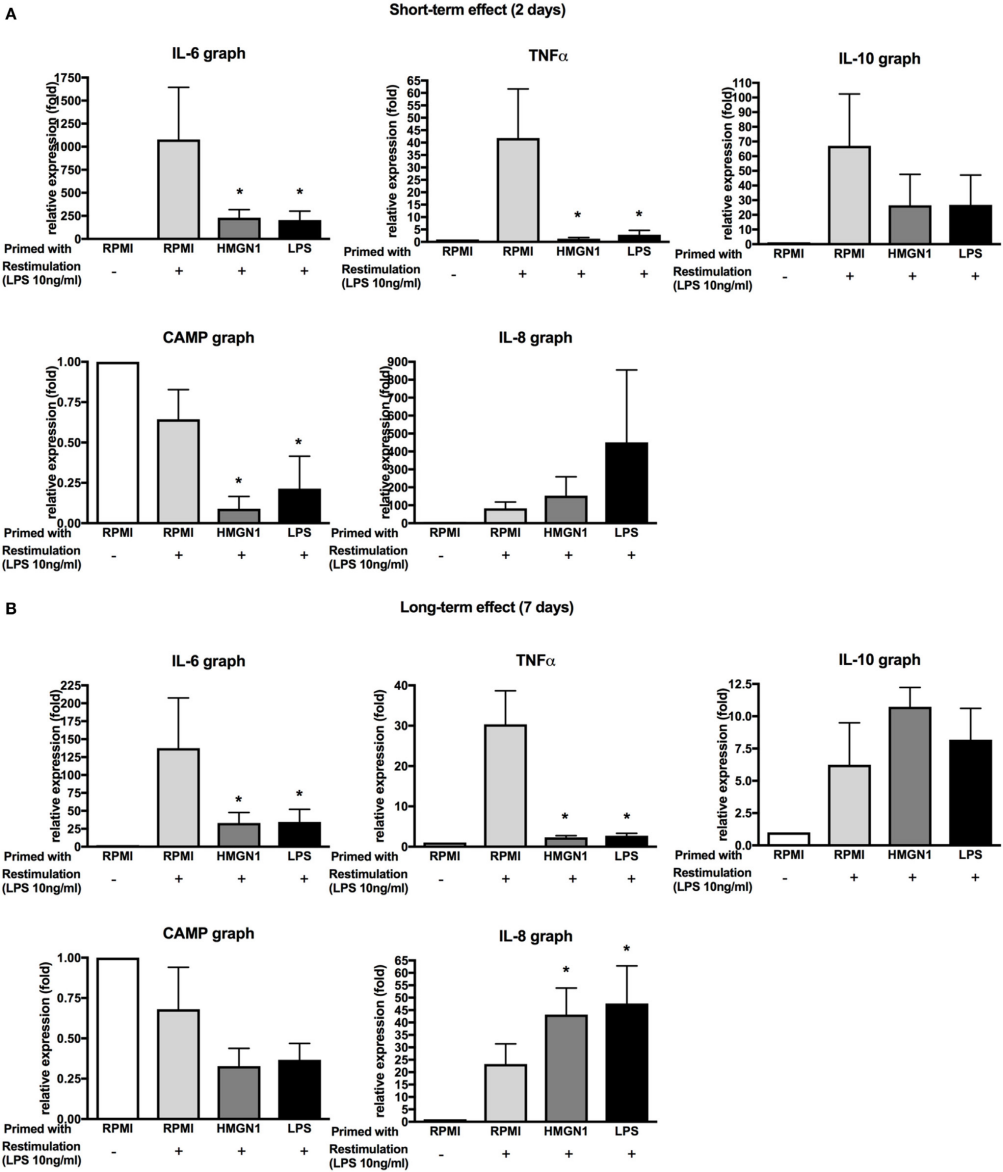
Effect of high-mobility group nucleosome-binding protein 1 (HMGN1) on TNF, IL-6, IL-8, IL-10, and CAMP production. Human peripheral blood mononuclear cells were primed with recombinant HMGN1 or lipopolysaccharide (LPS) for 24 h and then washed with PBS. The cells were further rested in RPMI containing 10% serum for **(A)** 24 h or **(B)** 7 days. The cells were stimulated with LPS or RPMI. The total RNA was extracted after 4 h and the different gene expression was measured by RT-PCR. The expression fold of target genes was normalized to the expression of HPRT [*n* = 5–6, *p < 0.05 vs RPMI (LPS restimulated) control].

Lipopolysaccharide priming has been demonstrated to induce transient silencing of pro-inflammatory mediators and priming of genes such as antimicrobial effectors (14). To address whether HMGN1 also induces similar differential gene regulation patterns, the mRNA expression of pro-inflammatory cytokines IL-6 and TNF-α, anti-inflammatory cytokine IL-10, chemokine IL-8 and antimicrobial peptide CAMP (cathelicidin-related antimicrobial peptide, also called LL-37) were determined by quantitative real-time PCR (Figure 5). In line with the cytokine results, pro-inflammatory cytokines TNF and IL-6 expression were significantly downregulated by both HMGN1 and LPS. IL-10 expression was downregulated by HMGN1 in the short-term incubations (albeit the difference was not significant) and recovered to the normal state in the long-term model. However, unlike the experiments earlier reported in mouse macrophages, neither in HMGN1- nor in LPS-induced tolerance could the expression of CAMP be induced in human PBMCs. Surprisingly, the expression of IL-8 was not inhibited, but was even enhanced after long-term incubation. The long-term effects of HMGN1 on IL-8 production were confirmed by ELISA (Figure S1 in Supplementary Material).

### HMGN1-Induced Immune Tolerance *In Vivo*

**Figure 6:**
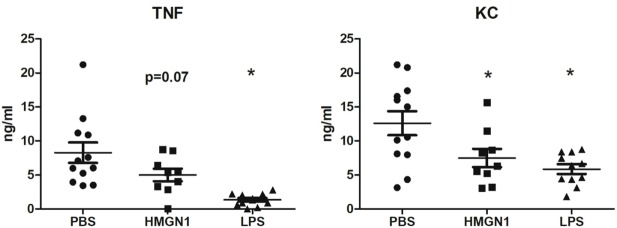
High-mobility group nucleosome-binding protein 1 (HMGN1)-induced tolerance *in vivo*. Mice were pretreated with HMGN1, lipopolysaccharide (LPS) or PBS intraperitoneally. After 6 h, mice were challenged with LPS for 1 h. TNF and KC level in serum were determined (*n* = 10–12, *p < 0.05).

To assess the pathophysiological role of HMGN1, mice were pretreated with either recombinant HMGN1 or LPS to induce tolerance for 6 h followed by secondary stimulatory LPS injection. TNF and KC production was significantly blunted in the LPS pretreated group compared to PBS control (Figure 6). Similarly, HMGN1 pretreatment also downregulated LPS-induced TNF and KC production, albeit the downregulated level was lower than that of LPS pretreated group.

### The Effects of Histone Methylation and Acetylation on HMGN1-Induced Tolerance

Epigenetic modifications have been suggested to play an important role for the LPS-induced tolerance through histone acetylation and methylation (18). To examine whether epigenetic modifications are also involved in HMGN1-induced tolerance, several enzymatic inhibitors of acetyl- and methyltransferases were added to the PBMCs prior to the priming stage. However, no obvious restoration of cytokine production was observed by the inhibitors we tested, with the exception of the short-term restoration effect induced by blocking histone acetylation by EGCG for IL-6 production (Figure S2 in Supplementary Material).

### The Effects on Sirtuin-1 and Immunometabolism by HMGN1 Stimulation

**Figure 7:**
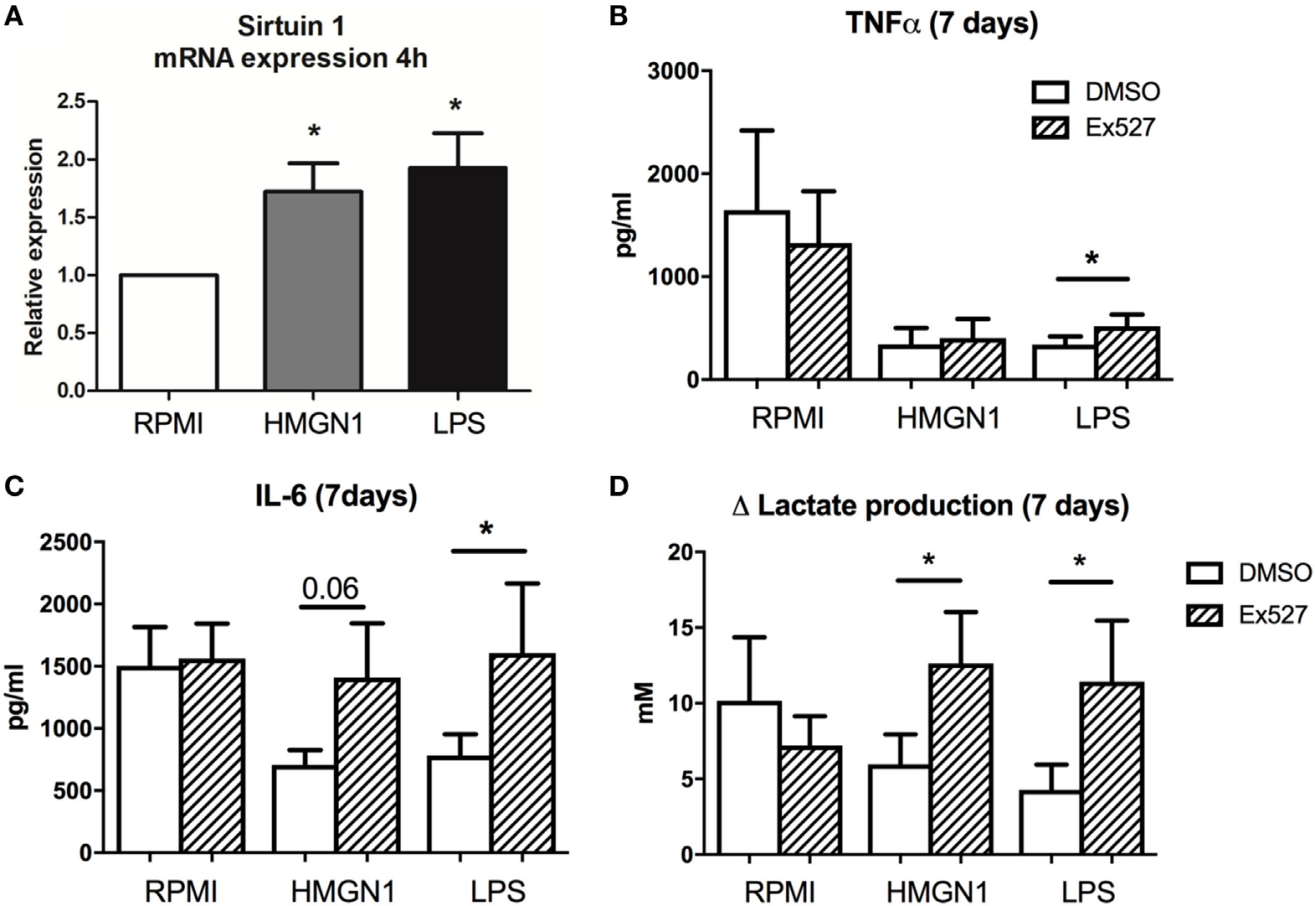
Inhibition of Sirtuin-1 restores cytokine and lactate production. Human peripheral blood mononuclear cells (PBMCs) were incubated with high-mobility group nucleosome-binding protein 1 (HMGN1) or lipopolysaccharide (LPS) for 4 h, then **(A)** mRNA was isolated and sirtuin-1 expression was determined by RT-PCR, showing an upregulation of sirtuin-1 for both stimuli. Human PBMCs were preincubated for 1 h with EX527 (sirtuin-1 inhibitor) before LPS or recombinant HMGN1 were added for 24 h. Then stimuli were washed away and cells rested in RPMI with 10% serum for an additional 6 days before they were restimulated with RPMI or LPS. **(B)** IL-6, **(C)** TNF, and **(D)** lactate production were assessed (*n* = 4–5, *p < 0.05).

It has been shown before that Sirtuin-1 (a histone deacetylation inhibitor) is a key regulator of LPS tolerance (17, 19). Sirtuin-1 has been shown to be upregulated during the early phase after LPS stimulation and has a driving role in the transition from a glycolytic energy metabolism to a more β-oxidation-dependent metabolism (19). First, Sirtuin-1 mRNA expression was upregulated after HMGN1 stimulation (Figure 7A). Second, the Sirtuin-1 inhibitor EX527 (17) partially restored cytokine production inhibited by HMGN1 (Figures 7B,C). Finally, EX527 also restored the capacity to release lactate after restimulation (as a measure of glycolysis) in both LPS- and HMGN1-tolerant macrophages (Figure 7D).

## Discussion

Although HMGN1 functions physiologically within the nucleus, the release of extracellular HMGN1 has been demonstrated to possess chemotactic function and to induce DC maturation (11). In the present study, we demonstrate that extracellular exposure of human PBMCs to HMGN1 induces a robust release of pro-inflammatory cytokines, such as IL-6 and TNF-α. This effect is likely to be relevant during sterile inflammation induced by perturbed cellular and/or tissue homeostasis (20), where the release of intracellular HMGN1 may cause acute local inflammation.

HMGB1 is the best-characterized HMG-family protein. It was initially identified as a nuclear protein that is important for the regulation of transcription (21). HMGB1 facilitates the binding of regulatory protein complexes to DNA by causing DNA bending (22) to enhance transcription activation (23). The extracellular HMGB1 was first described to be a late-acting mediator of endotoxemia and sepsis (24–26). Moreover, increasing evidence suggests the involvement of endogenous alarmins in ischemia–reperfusion injury (27, 28). The endogenous TLR4 ligands, such as HMGB1 and heat shock protein, have been demonstrated to be involved in these sterile inflammatory conditions (29, 30). In the human primary mononuclear cells used in the present study, we demonstrated that, similar to HMGB1, HMGN1 is a potent pro-inflammatory cytokine inducer and functions as an endogenous TLR4 ligand (11, 12).

Since LPS is well known to induce tolerance in monocytes through TLR4, we further examined whether HMGN1 could also induce similar tolerance effects. We demonstrated that HMGN1 is able to induce both short-term and long-term tolerance in terms of pro-inflammatory cytokines production in response to second stimulation with both TLR agonists as well as the dectin-1 ligand β-glucan. By blocking TLR4 signaling, the tolerance effect could be partially restored, indicating that HMGN1-induced tolerance is TLR4 dependent. In addition, intraperitoneal injection of HMGN1 into mice also renders the mouse less responsive to subsequent LPS stimulation, similar to LPS-mediated immune tolerance *in vivo*.

Earlier studies have shown that histone modifications play an important role in mediating the tolerance effects induced by LPS (14). We hypothesized that the long-term effects of HMGN1 effects may also be induced by epigenetic mechanisms. The involvement of epigenetic modulators for HMGN1-induced tolerance was examined using inhibitors of epigenetic modifier enzymes including ITF2357 (Histone deacetylase inhibitor), EGCG (Epigallocatechin-3-gallate, histone acetytransferase inhibitor) and pargyline (histone demethylase inhibitor). Only EGCG had a marginal effect on the short-term effects of HMGN1-induced tolerance. By contrast, a different picture emerged when the effect of the NAD+-dependent histone deacetylase Sirtuin-1 was studied (18). First, HMGN1 induced, just as LPS, Sirtuin-1 expression. Second, inhibition of Sirtuin-1 by a specific inhibitor partially restored cytokine production during HMGN1-induced tolerance. This provides further support for the sharing of the tolerance pathway by endotoxin and HMGN1. Sirtuin-1 is a pivotal downstream signal of this pathway.

An additional interesting observation concerns the interplay between immune activation of the cells and the cellular metabolism of glucose. A recent study demonstrated that induction of aerobic glycolysis (Warburg effect) is necessary for the effective production of cytokines by macrophages during LPS stimulation (31). Moreover, we have also recently reported that during trained immunity, a process mirroring tolerance that is also mediated by epigenetic reprogramming, namely induction of aerobic glycolysis is crucial (32). In line with this, the data presented here show that tolerant cells (both induced by HMGN1 or LPS) are not able to mount aerobic glycolysis, as mirrored by defective lactate production. Interestingly, the Sirtuin-1 inhibitor EX527 restored the capacity of monocytes to respond with lactate production upon stimulation with LPS, demonstrating that histone acetylation controls both immune and metabolic function of tolerant monocytes. This suggests Sirtuin-1 to be an attractive potential therapeutic target in immune tolerance and paralysis during Gram-negative sepsis and other severe infections.

In conclusion, HMGN1 induces tolerance in human PBMCs through a TLR4/Sirtuin-1 dependent mechanism, arguing that it may contribute to modulation of sterile inflammation in processes, such as severe trauma and ischemia-reperfusion, during which high amounts of TNF and IL-6 are released in the absence of exogenous stimuli (33). The sterile inflammation may be caused by the release of endogenous HMGN1 from the damaged cells and the induction of cytokines through TLR4 signaling. Moreover, the acute inflammation induced by HMGN1 might later translate into tolerance and even immunoparalysis, to increase the susceptibility of the patient to secondary infections. Therefore, blocking these HMGN1 effects may have potential therapeutic benefits in pathological processes in which hyperinflammation and/or immune paralysis play a role in pathogenesis.

## Ethics Statement

This study was carried out in accordance with the National Institute of Health Guide for the Care and Use of Laboratory Animals, with the approval of the Institutional Animal Care and Use Committee of National Tsing Hua University (Approval number: 10530, Hsinchu, Taiwan).

## Author Contributions

Conception and drafting of the article: RA, MN, and S-CC. Performed and analysis of experiments: RA and P-KH. Discussions of the data and critical revision of the article: RA, DY, LJ, JM, JO, MN, and S-CC.

## Conflict of Interest Statement

The authors declare that the research was conducted in the absence of any commercial or financial relationships that could be construed as a potential conflict of interest.
