# Supplementary material for: High-Mobility Group Nucleosome-Binding Protein 1 as Endogenous Ligand Induces Innate Immune Tolerance in a TLR4-Sirtuin-1 Dependent Manner in Human Blood Peripheral Mononuclear Cells
Source: Front Immunol. 2018 Mar 14;9:526. doi: 10.3389/fimmu.2018.00526 (PMC5861144; doi:10.3389/fimmu.2018.00526)
Supplement: Supplementary file 1 [file data_sheet_1.docx]

**Supplementary Figure 1**

**Supplementary Figure 1 -** Human PBMCs were incubated with HMGB1, HMGN1 or LPS for 24 hours. The cells were further resting in RPMI containing 10% serum for (A) 24 hours or (B) 7 days then stimulated with LPS or RPMI for additional 24 hours. Supernatant was harvested after 24 hours stimulation. The IL-8 level was determined by ELISA. (n=5-7).

**Supplementary Figure 2**

**
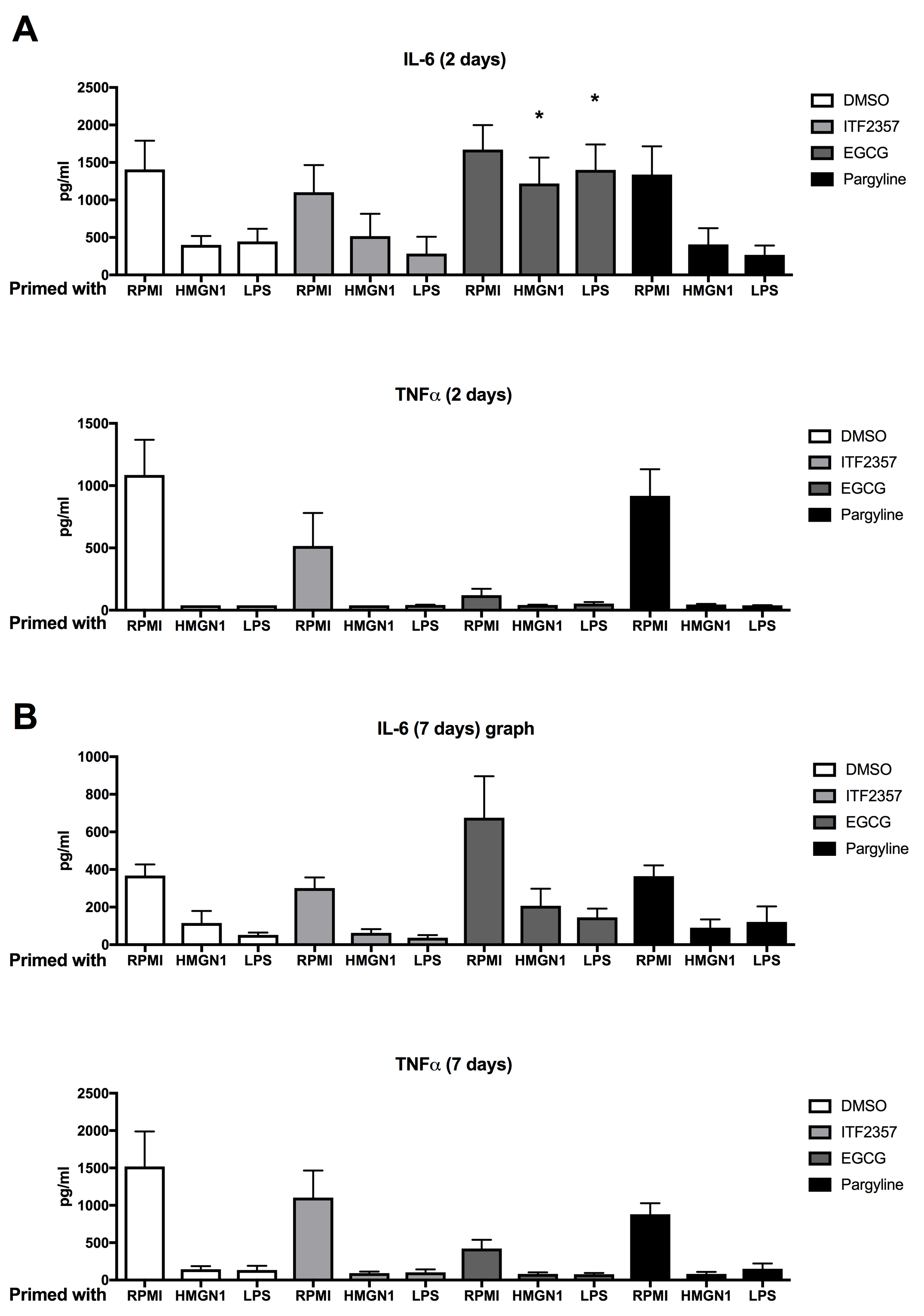
**

**Supplementary Figure 2 – Epigenetic inhibitors have no effect on HMGN1 induced tolerance.** Human PBMCs were preincubated with ITF2357, EGCG, pargyline or DMSO for 1 hours and then primed with recombinant HMGN1 or LPS for 24 hours. The cells were further resting in RPMI containing 10% serum for (A) 24 hours or (B) 7 days then stimulated with LPS or RPMI for additional 24 hours. Supernatant was harvested after 24 hours stimulation. The IL-6 and TNF-α level were determined by ELISA. (n =4-8, * p < 0.05 vs DMSO control).

**Supplementary Table 1**

qPCR primers:

| **Primer** | **Forward** **5’-> 3’** | **Reverse 5’-> 3’** |
| --- | --- | --- |
| IL-6 | AATTCGGTACATCCTCGACGG | GGTTGTTTTCTGCCAGTGCCT |
| IL-8 | ACTGAGAGTGATTGAGAGTGGAC | AACCCTCTGCACCCAGTTTTC |
| IL-10 | CAACCTGCCTAACATGCTTCG | TCATCTCAGACAAGGCTTGGC |
| TNF | CCTCTCTCTAATCAGCCCTCTG | GAGGACCTGGGAGTAGATGAG |
| CAMP | AGGTCCTCAGCTACAAGGAAG | TCTTGAAGTCACAATCCTCTGGT |
| Sirtuin 1 | TAGCCTTGTCAGATAAGGAAGGA | ACAGCTTCACAGTCAACTTTGT |
| HPRT | CCTGGCGTCGTGATTAGTGAT | AGACGTTCAGTCCTGTCCATAA |
